# Integrating a Low-Cost Electronic Nose and Machine Learning Modelling to Assess Coffee Aroma Profile and Intensity

**DOI:** 10.3390/s21062016

**Published:** 2021-03-12

**Authors:** Claudia Gonzalez Viejo, Eden Tongson, Sigfredo Fuentes

**Affiliations:** Digital Agriculture Food and Wine Group, School of Agriculture and Food, Faculty of Veterinary and Agricultural Sciences, University of Melbourne, Parkville, VIC 3010, Australia; eden.tongson@unimelb.edu.au (E.T.); sfuentes@unimelb.edu.au (S.F.)

**Keywords:** coffee aroma, quality traits, electronic nose, machine learning

## Abstract

Aroma is one of the main attributes that consumers consider when appreciating and selecting a coffee; hence it is considered an important quality trait. However, the most common methods to assess aroma are based on expensive equipment or human senses through sensory evaluation, which is time-consuming and requires highly trained assessors to avoid subjectivity. Therefore, this study aimed to estimate the coffee intensity and aromas using a low-cost and portable electronic nose (e-nose) and machine learning modeling. For this purpose, triplicates of nine commercial coffee samples with different intensity levels were used for this study. Two machine learning models were developed based on artificial neural networks using the data from the e-nose as inputs to (i) classify the samples into low, medium, and high-intensity (Model 1) and (ii) to predict the relative abundance of 45 different aromas (Model 2). Results showed that it is possible to estimate the intensity of coffees with high accuracy (98%; Model 1), as well as to predict the specific aromas obtaining a high correlation coefficient (R = 0.99), and no under- or over-fitting of the models were detected. The proposed contactless, nondestructive, rapid, reliable, and low-cost method showed to be effective in evaluating volatile compounds in coffee, which is a potential technique to be applied within all stages of the production process to detect any undesirable characteristics on–time and ensure high-quality products.

## 1. Introduction

Coffee accounted for 52% of total volume, considering the hot drinks category in 2020 and is expected to grow 3.61% by 2021. However, a decrease of 2.4% was observed in 2020 compared to 2019 due to the COVID-19 pandemic [1]. The total volume of coffee consumption in 2020 can be attributed to at-home consumption alone, which has grown during the worldwide lockdown. As an example of this phenomena, in Australia, in March 2020, during the pandemic, the at-home consumption increased by 37%, with the coffee beans being the type with the highest consumption growth (49%), followed by instant premium coffee (48%), while coffee capsules or pods accounting for 23% growth [2].

Aromas and flavors are among the most important sensory attributes that consumers consider when assessing coffee quality and liking [3,4,5]. These are mainly attributed to the coffee variety, provenance, as well as roasting process, including time and temperature [4,6,7]. Due to the differences derived from the provenance factor in the coffee attributes, especially aromas that are major contributors to the coffee quality, producers have opted to certify their products; this process ensures that consumers relate to the specific aromas of the coffee associated with their origin, quality perception and, hence, contributes to higher acceptability [7,8]. There are also different factors in the coffee brewing that may affect the aroma in the final product; some of these are water temperature and hardness, as well as the method or machine used. In the case of water temperature, if it is not hot enough (ideal: 85–95 °C), volatile aromatic compounds are not fully incorporated and released, providing a coffee that is weak and low in aromatics [9]. Regarding water hardness, Dadali et al. [10] found that coffee brewed with medium water hardness (Total dissolved solids = TDS = 141 mg L^−1^) provides a product with more aromas compared to soft (TDS = 60 mg L^−1^) and high water hardness (TDS = 424 mg L^−1^). Caprioli et al. [11] mentioned several studies in which different aroma profiles have been found for distinct preparation methods such as boiled coffee brewing and pressurized espresso brewing.

To ensure that the final products (brewed coffee) will be acceptable by consumers, different techniques are used to assess their quality traits. Traditional methods to assess aromas in coffee consist of either instrumental techniques using gas-chromatography/mass-spectroscopy (GC/MS) [12,13], or most commonly through sensory analysis using descriptive sensory panels [14] and methods such as quantitative descriptive analysis (QDA^®^), or expert panels. The latter tend to be less reliable as sensory panels are often subjected to more biases, such as the habituation error [15]. Furthermore, these techniques tend to be time-consuming, are destructive, and require larger sample sizes; they involve higher costs and high expertise for data acquisition, analysis and interpretation [5,6,16,17,18,19,20].

Electronic noses (e-noses) were first designed and proposed in the early 1980s by Persaud and Dodd [21], who developed an e-nose using semiconductor transducers and finding that this was able to discriminate a broad range of odors. Following this, some researchers have either developed or used commercial e-noses as an alternative to traditional methods to assess aromas or other chemometrics in food and beverages such as beer [17,22,23,24], wine [16,25], meat [26], juices [27,28], saffron [29], and tea [30,31,32], among others. Likewise, e-noses have been used in coffee to assess aromas and predict sensory descriptors using artificial neural networks (ANN) [33], to predict the geographical origin using discriminant factorial analysis [4], to discriminate between civet and non-civet coffee [5,34], and to predict the roasting degree using ANN [35], among others. However, most of the e-noses used in the aforementioned studies are non-portable, and most of them, despite having lower costs than GC/MS, are still cost-prohibitive for small and medium companies.

The use of machine learning (ML) has been applied to different industries such as sustainability of materials [36], techno-economics [37], molecular crystals engineering [38], energy [39], diagnostics in medicine [40] and, more recently, food/beverages [17,18,22,29,41] and agriculture [42,43,44]. This has been an effective tool to aid in the prediction and rapid assessment of products; however, a common issue found when using ML is the overfitting of the models because the generalization of the data is not achieved. This is usually found in noisy datasets and when the number of selected neurons is too high, which provides high accuracy, but poor performance [45,46]. Among the supervised ML algorithms, artificial neural networks tend to be the most robust due to their nonlinearity and good capacity of finding patterns among the inputs and targets. Furthermore, this is most convenient when a single multitarget model is required, which is a feature that is not possible in other ML algorithms [47,48]. Some studies that have presented multitarget ANN models include the identification of proteomics from beer foamability analysis [49], prediction of beer aromas and physicochemical data using foam-related parameters obtained from a robotic pourer [50], prediction of beer acceptability of sensory attributes using e-nose data [22], and prediction of sensory profiles of wine using near-infrared spectroscopy [51].

This study aimed to predict coffee aromas and roasting intensity using a newly developed low-cost and portable (wireless) e-nose coupled with machine learning (ML) modeling. Model 1 was developed using the e-nose data as inputs to classify samples into low-, medium-, and high-intensity according to that reported in their label. On the other hand, Model 2 was developed using the e-nose outputs as inputs to predict the relative abundance based on the peak area of 45 aromas measured using GC/MS used as targets.

By adding low-cost sensor technology with coffee pouring devices or normal coffee machines, the aroma profile from specific coffee and provenance can be maintained as a quality assurance method. It would also offer an automated system to detect aroma profile variations due to unforeseen factors, such as water quality, temperature or other problems in the brewing process that may affect consumer perception.

## 2. Materials and Methods

### 2.1. Samples Description

Samples used in this study consisted of nine coffees from Nespresso^®^ pods (Nestlé Nespresso S.A., Lausanne, Switzerland) with different intensities (Table 1). All samples were measured in triplicates (three pods) and brewed in a Creatista Plus Breville machine (Breville Group Ltd., Sydney, NSW, Australia) using a constant pouring volume of 110 mL at 78 °C.

### 2.2. Electronic Nose Description and Data Extraction

A low-cost and portable e-nose developed by the Digital Agriculture Food and Wine Group from The University of Melbourne (DAFW; UoM) and composed of nine different gas sensors (Henan Hanwei Electronics Co., Ltd., Henan, China; Table 2) was used to assess the coffee samples as described by Gonzalez Viejo et al. (2020). Modifications to the method consisted of the time of calibration (30 s before and 30 s after measurements) and time of exposure to each sample (1 min).

**Figure 1:**
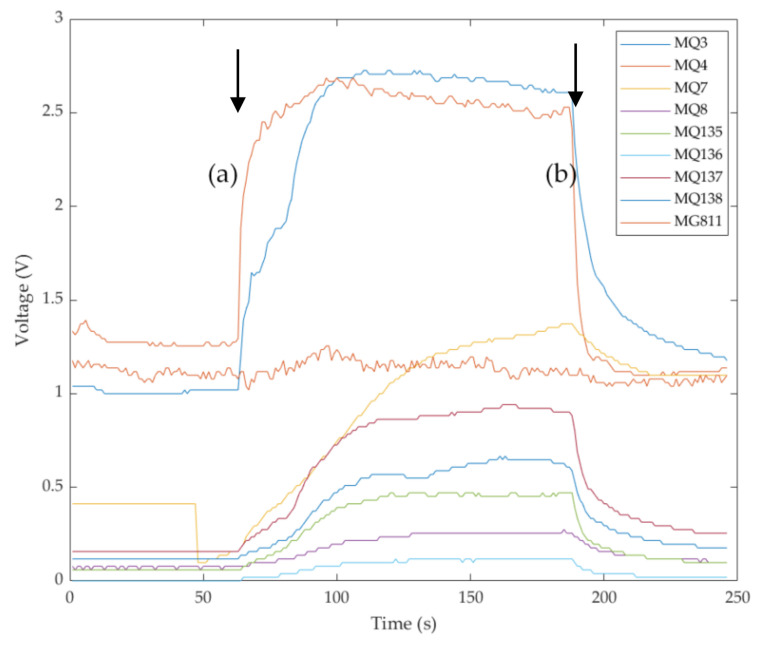
Data extraction from e-nose outputs using a supervised customized code written in Matlab. Initial (**a**) and final (**b**) stable signal detection and automatic subdivision in ten equidistant sampling intervals to obtain ten averages.

Data extraction was performed using a supervised automatic code written in Matlab^®^ R2020b (Mathworks Inc., Natick, MA, USA) to recognize stable e-nose signals within each coffee pouring (beginning and endpoints). From the initial and endpoints detected (Figure 1), ten subdivisions of the e-nose data are automatically performed to extract average values per sensor, which are used as inputs for the ML modeling.

### 2.3. Gas Chromatography-Mass Spectroscopy

The GC/MS method was used to identify volatile compounds found in the coffee samples and their relative abundance to be used as targets to further develop the ML model. For this purpose, the same samples measured using the e-nose were assessed for volatile aromatic compounds using a gas chromatograph with mass selective detector 5977B (GC/MSD; Agilent Technologies, Inc., Santa Clara, CA, USA; detection limit 1.5 fg). This was coupled with a PAL3 autosampler system (CTC Analytics AG, Zwingen, Switzerland) to measure the entire batch of samples in a single run. A total of 5 mL of each coffee sample replicate was placed in a 20 mL using an 18 mm magnetic screw cap with polytetrafluoroethylene and silicone septum. To ensure no carryover effects, two blank samples were included, one at the start and one at the end of the batch of samples. The method used was as described by Gonzalez Viejo et al. (2019). An HP-5MS (Agilent Technologies, Inc., Santa Clara, CA, USA) column and headspace method with solid-phase microextraction (SPME) divinylbenzene–carboxen–polydimethylsiloxane (DVB–CAR–PDMS) fiber (Agilent Technologies, Inc., Santa Clara, CA, USA) were used. Furthermore, helium was used as the carrier gas at a flow rate of 1 mL min^−1^. The inlet was set to splitless mode.

For volatile compounds identification, the National Institute of Standards and Technology library (NIST; National Institute of Standards and Technology, Gaithersburg, MD, USA) was used. For this, only the compounds identified with a certainty > 80% were reported. Compounds with a very low relative abundance and that were only present in one or two samples were removed for the purposes of this study.

### 2.4. Statistical Analysis

An analysis of variance (ANOVA) was conducted using the data from e-nose and GC–MS to assess significant differences (p <0.05) among samples and least significant difference (LSD) post hoc test for pairwise comparisons (α = 0.05) using XLSTAT v.2020.3.1 (Addinsoft, New York, NY, USA). Furthermore, a multivariate data analysis based on principal component analysis (PCA) was conducted using the volatile aromatic compounds’ peak area and constructed with Matlab^®^ R2020b to assess relationships among some of the variables and identify associations with the samples. Furthermore, the PCA was developed to assess groupings among the samples according to the roasting intensity level. The principal components (PC) one and two were selected based on them summing > 60% of data variability, which is the cutoff point considered to test the significance of the PCA [52]. Factor loadings (FL) of the most representative variables of each PC were obtained.

**Figure 2:**
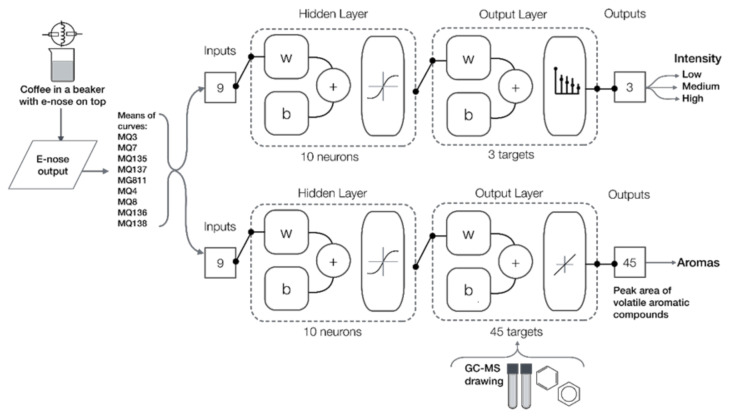
Diagram of the pattern recognition Model 1 (top) to classify samples into low-, medium-, and high-intensity, and regression Model 2 (bottom) to predict the peak area of 45 volatile aromatic compounds using the outputs from the electronic nose as inputs for both models. Abbreviations: b: bias; W: weights; sensors descriptions are presented in Table 2. Targets/outputs from Model 2 can be found in Table 3 (Results and Discussion).

A total of 25 supervised ML classifiers available in Matlab^®^ R2020b Classification Learner in the Statistics and Machine Learning Toolbox 12.0, which consist of decision trees (three algorithms), discriminant analysis (two algorithms), logistic regression (one algorithm), naïve Bayes (two algorithms), support vector machine (SVM; six algorithms), k-nearest neighbor classifiers (KNN; six algorithms), and ensemble classifiers (five algorithms, were tested (data not shown). Along with this, 17 artificial neural network (ANN) algorithms [47,53] were tested using a code written in Matlab^®^ R2020b to assess all different training algorithms in a loop and find the most accurate models based on the correlation coefficient/accuracy and performance. For Model 1, the best results were obtained using pattern recognition ANN with the mean values of ten sections of the highest and stable segment of each sensor’s curve as inputs to classify samples into i) low (3–5), ii) medium (6–9), and iii) high (10–13) intensity (Figure 2). For this model, the Bayesian regularization training algorithm resulted in the highest accuracy and no overfitting signs. Data division was random, with 70% of the samples used for training and 30% for testing using a means squared error (MSE) performance algorithm. This is a two-layer feedforward model with a tan-sigmoid function in the hidden layer and a Softmax function in the output layer. Statistical data reported for this model consists of accuracy (%), error (%), MSE values and receiver operating characteristics (ROC) curve.

For Model 2, which was a regression model, only the 17 ANN supervised training algorithms [47,53] were tested as other ML techniques do not allow multiple targets. This model consisted of using the mean values of ten sections of the highest and stable segment of each sensor’s curve as inputs to predict 45 volatile aromatic compounds (Figure 2). The Levenberg–Marquardt training algorithm was selected as the best based on the highest correlation coefficient (R) and performance with no overfitting signs. Data were divided randomly as 70% for training, 15% for validation using an MSE performance algorithm, and 15% for testing. For both models, a neuron trimming test (3, 5, 7, and 10 neurons) was performed to select the models with no under- or overfitting signs, resulting in 10 the optimal number of neurons for the two models. This is a two-layer feedforward model with a tan-sigmoid function in the hidden layer and a linear transfer function in the output layer. Statistical data reported for this model consists of R, slope, MSE values and the overall regression model figure.

The samples used for both models consisted of the number of different coffees (nine) times the number of replicates (three) times the number of means from the e-nose curves (ten), which equals 270 samples. A similar approach has been used in previous studies [16,17]. The number of samples used for the models is sufficient, considering that the dataset is small enough to avoid having enough power to overfit the model [53,54].

## 3. Results and Discussion

**Figure 3:**
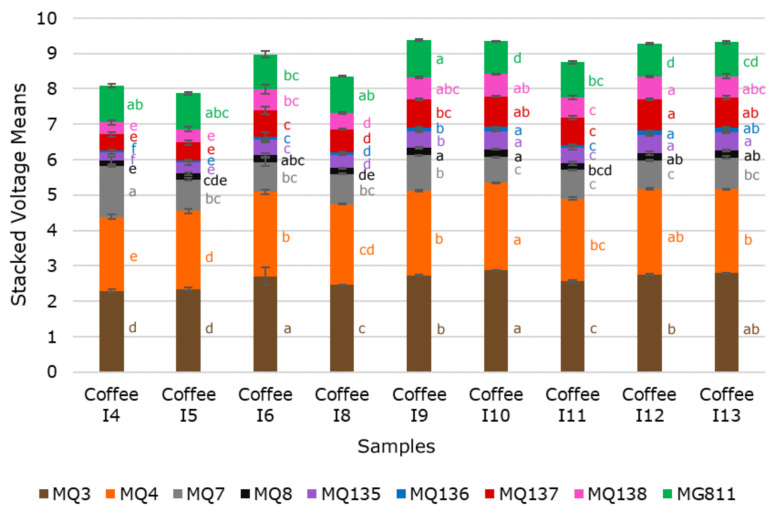
Stacked mean values of the electronic nose sensors showing the letters of significance that depict significant differences between samples according to the ANOVA and least significant differences (LSD) post hoc test (*p* < 0.05; α = 0.05). Differences are compared for each sensor among samples (bar colors). Samples and sensors description can be found in Table 1 and Table 2, respectively. Error bars represent the standard error.

Figure 3 shows significant differences (*p* < 0.05) between samples in all sensors from the e-nose. It can be observed that the sensors with the highest voltage for all samples were MQ3 and MQ4 sensors, being Coffee I10 with the highest voltage. A study conducted using civet and non-civet coffee beans and measured using an e-nose that included some of the sensors in the present paper reported MQ7 sensor (Carbon monoxide (CO)) as the highest voltage followed by MQ3 (ethanol) and MQ4 [34]. The authors of the mentioned paper did not specify the conditions in which the measurements were performed; however, the fact that there is CO production during the roasting process of coffee beans [55] may explain their high levels in the MQ7 sensor. Therefore, the lower voltage associated with MQ7 in the samples presented in this paper may be associated with the brewing process.

Table 3 shows the 45 identified volatile aromatic compounds using the GC/MS and the associated aromas. Peak area and retention times, as well as ANOVA-LSD results for each compound and sample, may be found in the Supplementary Material (Table S1). There were non-significant differences (*p* > 0.05) between samples for compounds C2, C5, C7, C8, 12, C27, and C31; all other compounds presented significant differences (*p* < 0.05) between the coffee samples. In Table 3, it can be observed that most of the volatile compounds are associated with coffee, cocoa, and nutty aromas, which have also been reported in other published studies, some of these are furans such as 2,5-dimethylfuran (C2), 2-methoxymethyl furan (C8), 2-acetyl furan (C12), 2,2-methylenebisfuran (C31), furfuryl propionate (C32); pyrazines such as 2-methylpyrazine (C7), 2-ethylpyrazine (C13), coffee pyrazine (C20), and nutty pyrazine (C36), among others [12,13,56]. The most abundant compound based on the peak area for all samples was furfuryl acetate (C18), which has been related to fruit, banana, and ethereal aromas (Table S1); other authors have also found this compound in arabica [57], and robusta [58] coffees and they have associated this compound also with a floral aroma. Some compounds related to smoke have also been found, some of these are pyridine (C4) and phenols such as ortho-guaiacol (C33), 4-ethyl guaiacol (C43), and 4-vinyl guaiacol (C45), these are formed during the roasting process of the coffee beans [58,59]. In this study, coffees with higher intensity presented larger peak areas for compounds C4, C33, C43, and C45, than lower intensities (Table S1). 3-Methylfuran (C1), which is considered a toxic, carcinogenic compound, was identified. This has been reported in studies from other authors and found to be formed during the roasting process of coffee [60,61]; however, the International Agency for Research on Cancer (IARC) has not found sufficient evidence of possible carcinogenic effects due to coffee consumption [62].

**Figure 4:**
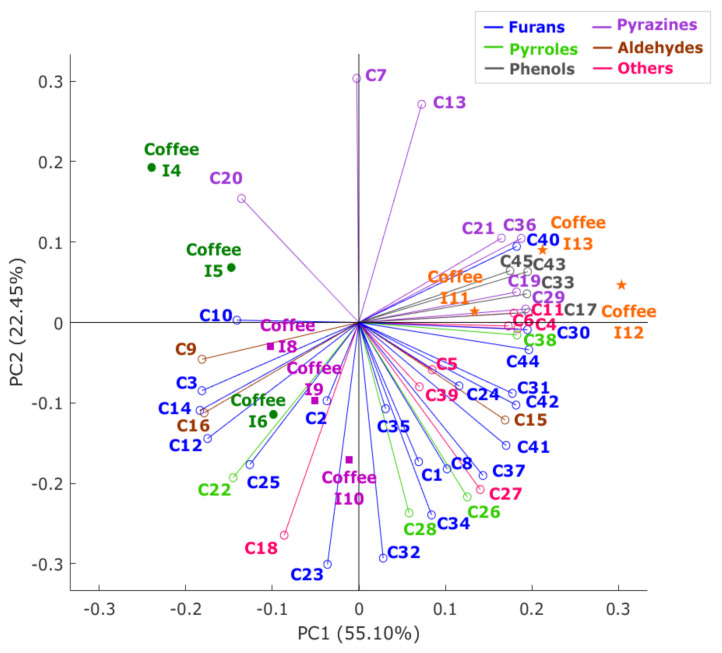
Principal component analysis biplot depicting the volatile aromatic compounds and coffee samples. Each vector color represents a functional group to which the compounds belong. Abbreviations of the volatile aromatic compounds can be found in Table 3 and samples in Table 1.

Figure 4 shows that principal components one and two (PC1 and PC2) represented 77.55% of the total data variability. According to the factor loadings (FL; Table S2), the PC1 was mainly represented by the phenols C17, and C43, and the furan C44, all with FL = 0.20 on the positive side of the axis, and by furans C3 and C14, and aldehyde C9, all with FL = −0.18, on the negative side. On the other hand, on the positive side of PC2, it was mainly characterized by pyrazines C7 (FL = 0.30) and C13 (FL = 0.27), while on the negative side, it was described by furans C23 (FL = −0.29) and C32 (FL = −0.30). It can be observed that coffees were mainly grouped by intensity levels, represented with different colors in the PCA. Contrary to all other samples, coffees of high-intensity levels (I11–I13) were mainly associated with phenols, which, as previously mentioned, are mainly the smoke aromas, which are formed during the roasting process.

**Figure 5:**
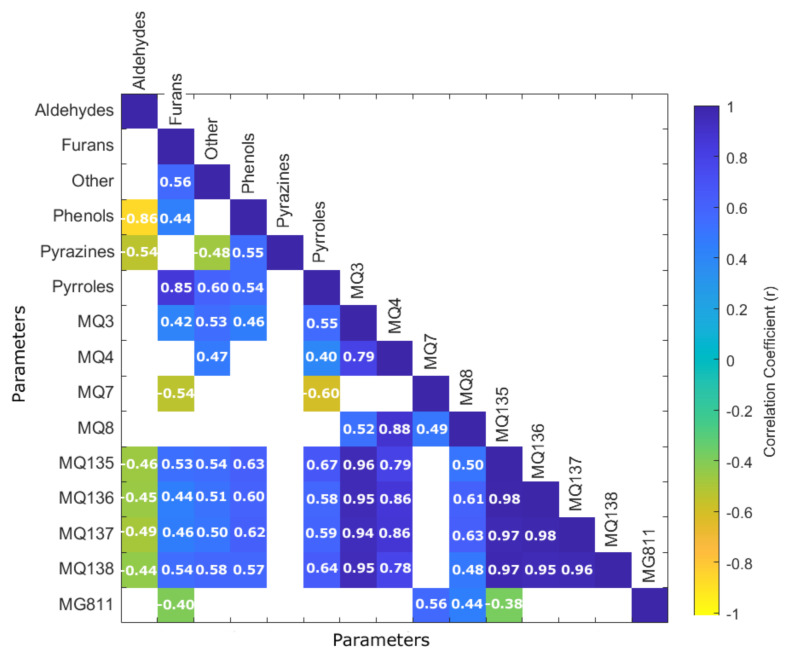
Matrix showing the significant correlations (*p* < 0.05) between the volatile aromatic compounds functional groups and the sensors integrated into the electronic nose. Correlation coefficients are depicted with different colors according to the color bar in which yellow represents the negative correlations, while blue shows the positive correlations.

Figure 5 shows the matrix with significant correlations (*p* < 0.05) between the volatile aromatic compounds grouped by functional groups from the GC–MS analysis and the results from the nine e-nose sensors. It can be observed that MQ3 sensor was positively correlated with furans (r = 0.42), phenols (r = 0.54), pyrroles (r = 0.55), and others (r = 0.60). The “others” group comprises compounds that belong to esters, pyridines, aromatic hydrocarbons, acetates, and ketones. Some compounds in the furans and other groups contain an alcohol functional group, explaining their correlation with the MQ3 sensor. Even though this sensor is most sensitive to alcohol, it also has a lower sensitivity to smoke, which may explain its correlation with phenols, as well as pyrroles and furans, which are composed of some compounds associated with smoke and burnt aromas [63]. MQ4 sensor was positively correlated with pyrroles (r = 0.40) and others (r = 0.47); this sensor is mainly sensitive to methane and butane but also has a lower sensitivity to alcohols and smoke, which may explain these correlations. On the contrary, MQ7 was negatively correlated with furans (r = −0.54) and pyrroles (r = −0.60). Furthermore, sensors MQ135, MQ136, MQ137 and MQ138 were negatively correlated with aldehydes (r = −0.44–−0.49) and positively correlated with furans (r = 0.44–0.54), phenols (r = 0.57–0.63), pyrroles (r = 0.58–0.67) and others (r = 0.50–0.58). Sensor MG811 was negatively correlated with furans (r = −0.40).

**Figure 6:**
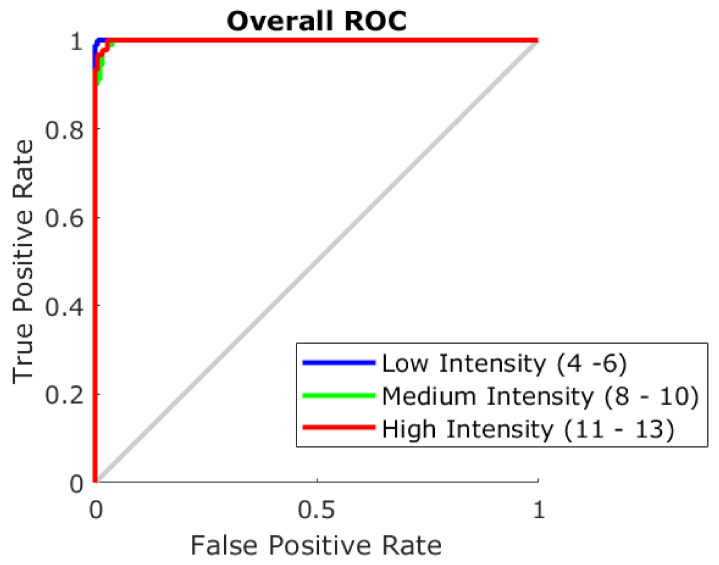
Overall receiver operating characteristics (ROC) curve showing the true positive (sensitivity; y-axis) and false-positive (specificity; x-axis) rates of the classification Model 1.

Given the correlations between the e-nose outputs and GC–MS results, two ML models were developed using the e-nose outputs as inputs, as described in Section 2.4. Table 4 shows the accuracy of Model 1 to predict coffees’ intensity level as low, medium, and high. It can be observed that the overall accuracy was 98% with training and testing stages resulting in 100% and 94%, respectively. The lower training MSE value (<0.01) compared to the testing stage (MSE = 0.04) confirms that there were no signs of under- or overfitting of the model. Figure 6 shows the overall receiver operating characteristics (ROC) curve in which the three categories are very close to the highest sensitivity (true positive rate).

**Figure 7:**
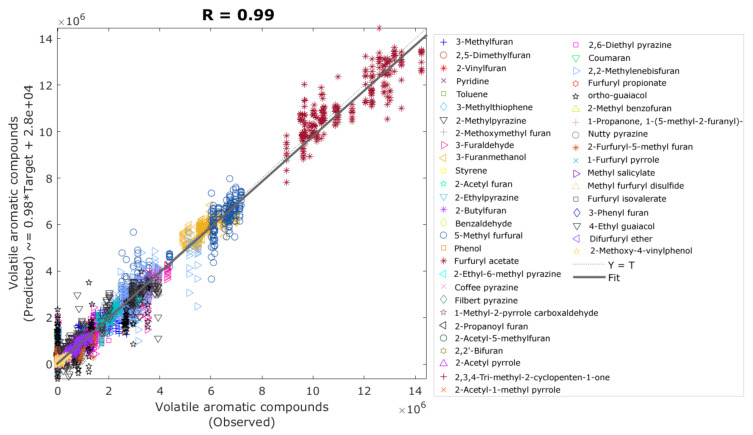
Overall artificial neural network regression model showing the observed (x-axis) and predicted (y-axis) values of the peak area of the volatile aromatic compounds (Model 2). Abbreviations: R: correlation coefficient, T: targets.

Table 5 shows that Model 2 had a very high accuracy in the training, validation and testing stages based on the correlation coefficient (R = 0.99, R = 0.98, and R = 0.99, respectively), with an overall accuracy R = 0.99. This, along with the lower training MSE value (6.3 × 1010) compared to the validation and testing stages, shows no signs of under- or overfitting. Furthermore, all stages presented a high slope close to the unity (b ~1). Figure 7 shows the overall regression model with each volatile aromatic compound depicted using a different marker and color, in which it can be observed that furfuryl acetate appears as the highest peak area, as confirmed in Table S1.

These models showed that the portable and low-cost e-nose coupled with ML is a reliable and effective tool to assess coffee intensity levels and the relative abundance of 45 different volatile aromatic compounds. Even though there are already published studies using e-noses to assess coffee aromas, and some also include the use of ML, they have been implemented in different ways. Michishita et al. [33] used an e-nose coupled with ANN to predict sensory descriptors; however, the authors used an αFOX4000 (Alpha M.O.S., Toulouse, France) commercial, non-portable and high-cost e-nose. Wakhid et al. [34] used a similar e-nose along with ML for a more specific and limited use to classify samples into civet or non-civet. The e-nose these authors presented only had five sensors that are sensitive to gases such as CO_2_, methane, CO, and natural gas; however, they did not include any sensor related to faulty aromas such as H_2_O, NH_3_ and aromatics such as benzene, which are included in the present paper and have shown to be effective in detecting more aromas in beverages such as beer and wine [16,17]. Romani et al. [35] presented a method to predict the roasting time of coffee using a PEN2 (Airsense Analytics, Milano, Italy) e-nose, which, although it claims to be portable, is still quite large and requires a bench, and consumables such as specific vials to function. Besides this, the authors presented an ANN regression model to predict the roasting time; however, their models, although with high determination coefficients (R^2^ > 0.96), only had eight data points, which is not sufficient for any regression model.

The method presented in this study has some advantages over other similar proposed techniques, as mentioned above. Further advantages include that the e-nose presented in this study is portable, wireless, and low-cost compared to other commercial e-noses and GC/MS equipment methods. Furthermore, the e-nose and models are validated with robust technology, such as the GC/MS, verifying its high accuracy and objective results. The fact that the e-nose is portable and fully wireless makes it more convenient for transporting the device and measuring samples in any location with the potential to be used in the field to be used to assess coffee beans. A disadvantage of the proposed method would be the need for GC/MS data used as ground-truth to develop further models, including additional samples with different characteristics to generate a more universal artificial intelligence model accounting coffee provenance, brands, water temperature and hardness effect, among others. However, once these further models have been developed using the methodology proposed in this paper, they will be more robust, cost-effective and accurate enough for repetitive sampling.

Among the potential applications of this method are that it would allow coffee producers and brewers to assess their products in a more efficient and affordable way and associate results with coffee quality based on its aroma, which may be measured in every batch of coffee. This would also be useful for manufacturers to identify the most abundant aromas that may be reported in their labels, which would be a more objective, less time-consuming, and rapid method rather than using trained sensory panels. Further studies should focus on the development of ML models to detect and identify faults.

## 4. Conclusions

The low-cost e-nose and ML models developed in this study can be integrated into coffee pod machines to assess potential changes to original aroma profiles and intensities due to water quality (hardness), different water temperatures or any other external factor affecting the brewing process. The system proposed can be added to commercial machines to secure specific quality and freshness of coffee grains related to provenance. Furthermore, this system can be used as quality control at the commercialization point for consumers to detect undesirable aromas. In this context, Model 1 would be useful to verify that the product obtained the desired roasted intensity level, while Model 2 is proposed to assess aromas in the coffee and ensure no undesirable compounds are present. Further studies will focus on using the low-cost and portable e-nose to assess coffee brewed under different conditions such as water temperature and hardness.

## Figures and Tables

**Table 1:** Samples used for the study, including their specifications and labels.

Sample	Intensity	Label	Variety
Volluto	4	Coffee I4	Arabica
Capriccio	5	Coffee I5	Arabica/robusta
Genova Livanto	6	Coffee I6	Arabica
Roma	8	Coffee I8	Arabica/robusta
Firenze Arpeggio	9	Coffee I9	Arabica
Ristretto Italiano	10	Coffee I10	Arabica/robusta
India	11	Coffee I11	Arabica/robusta
Palermo Kazaar	12	Coffee I12	Arabica/robusta
Napoli	13	Coffee I13	Arabica/robusta

**Table 2:** Sensors included in the electronic nose and the gases to which they are sensitive.

Sensor Name	Gas
MQ3	Ethanol
MQ4	Methane (CH_4_)
MQ7	Carbon monoxide (CO)
MQ8	Hydrogen (H)
MQ135	Ammonia/alcohol/benzene
MQ136	Hydrogen sulfide (H_2_S)
MQ137	Ammonia (NH_3_)
MQ138	Benzene/alcohol/ammonia
MG811	Carbon dioxide (CO_2_)

**Table 3:** Identified compounds from the gas chromatography mass-spectroscopy analysis, functional group, and their associated aromas.

Label	Compound	Functional Group	Aroma *	Label	Common Name	Functional Group	Aroma *
**C1**	3-Methylfuran	Furans	Toxic compound	**C24**	2-Acetyl-5-methylfuran	Furans	Musty/nutty/coconut/milky
**C2**	2,5-Dimethylfuran	Furans	Meaty/coffee/chocolate	**C25**	2,2’-Bifuran	Furans	Medicinal/camphor
**C3**	2-Vinylfuran	Furans	Phenolic coffee grounds	**C26**	2-Acetyl pyrrole	Pyrroles	Cherry/licorice/walnut/bready
**C4**	Pyridine	Other	Sour/smoke/coffee/burnt	**C27**	2,3,4-Trimethyl-2-cyclopenten-1-one	Other	Naturally found in *Jatropha ribifolia*
**C5**	Toluene	Other	Sweet	**C28**	2-Acetyl-1-methyl pyrrole	Pyrroles	Earthy/nutty/smoke/musty
**C6**	3-Methylthiophene	Other	Fatty/winey	**C29**	2,6-Diethyl pyrazine	Pyrazines	Nutty/hazelnut
**C7**	2-Methylpyrazine	Pyrazines	Nutty/cocoa/roasted/peanut	**C30**	Coumaran	Furans	Green tea
**C8**	2-Methoxymethyl furan	Furans	Roasted coffee	**C31**	2,2-Methylenebisfuran	Furans	Roasted/coffee
**C9**	3-Furaldehyde	Aldehydes	Coconut	**C32**	Furfuryl propionate	Furans	Banana/coffee/spicy
**C10**	3-Furanmethanol	Furans	Burnt/tobacco	**C33**	ortho-guaiacol	Phenols	Smoke/spice/vanilla/wood
**C11**	Styrene	Other	Sweet/balsam/resin	**C34**	2-Methyl benzofuran	Furans	Phenolic/burnt
**C12**	2-Acetyl furan	Furans	Almond/cocoa/coffee/roasted	**C35**	1-Propanone, 1-(5-methyl-2-furanyl)-	Furans	Green/hazelnut
**C13**	2-Ethylpyrazine	Pyrazines	Nutty/roasted/cocoa/coffee	**C36**	Nutty pyrazine	Pyrazines	Earthy/nutty/coffee/roasted
**C14**	2-Butylfuran	Furans	Wine/sweet/spicy/fruity	**C37**	2-Furfuryl-5-methyl furan	Furans	Naturally found in coffee
**C15**	Benzaldehyde	Aldehydes	Almond/cherry	**C38**	1-Furfuryl pyrrole	Pyrroles	Coffee/bready/mushroom
**C16**	5-Methyl furfural	Aldehydes	Spice/caramel/bready/coffee	**C39**	Methyl salicylate	Other	Wintergreen mint
**C17**	Phenol	Phenols	Phenolic/plastic/rubber	**C40**	Methyl furfuryl disulfide	Furans	Roasted coffee/sulfur/meaty
**C18**	Furfuryl acetate	Other	Fruity/banana/ethereal	**C41**	Furfuryl isovalerate	Furans	Berry/grape/plum
**C19**	2-Ethyl-6-methyl pyrazine	Pyrazines	Roasted potato/roasted hazelnut	**C42**	3-Phenyl furan	Furans	Cocoa/green/minty
**C20**	Coffee pyrazine	Pyrazines	Coffee bean/nutty/roasted	**C43**	4-Ethyl guaiacol	Phenols	Spicy/smoky/clove
**C21**	Filbert pyrazine	Pyrazines	Nutty/musty/earthy/bready	**C44**	Difurfuryl ether	Furans	Coffee/nutty/earthy/mushroom
**C22**	1-Methyl-2-pyrrole carboxaldehyde	Pyrroles	Roasted/nutty	**C45**	4-Vinyl guaiacol	Phenols	Woody/roasted/peanut/smoke
**C23**	2-Propanoyl furan	Furans	Fruity				

* Associated aromas were obtained from The Good Scents Company [63].

**Table 4:** Results from the artificial neural network pattern recognition model (Model 1) to classify coffee samples according to the intensity level, showing the accuracy and error of each stage. Performance was assessed based on mean squared error (MSE).

Stage	Samples	Accuracy	Error	Performance (MSE)
Training	189	100%	0%	<0.01
Testing	81	94%	6%	0.04
Overall	270	98%	2%	-

**Table 5:** Statistical data of the artificial neural network regression model (Model 2) to predict the peak area of volatile aromatic compounds. Performance was assessed based on mean squared error (MSE). Abbreviations: R: correlation coefficient.

Stage	Samples	Observations	R	Slope	Performance (MSE)
Training	188	8460	0.99	0.98	6.3 × 10^10^
Validation	41	1845	0.98	0.97	14.2 × 10^10^
Testing	41	1845	0.99	0.97	11.2 × 10^10^
Overall	270	12,150	0.99	0.98	-

## Data Availability

Data and intellectual property belong to The University of Melbourne; any sharing needs to be evaluated and approved by the University.

## Supplementary Materials

The following are available online at https://www.mdpi.com/1424-8220/21/6/2016/s1, Table S1. Identified compounds from the gas chromatography mass-spectroscopy analysis showing the mean values of the peak area. Table S2. Factor loadings of the main two components of the principal component analysis. Abbreviations of the compounds are found in Table 3; PC1 and PC2: Principal components one and two.
