# Supplementary material for: Integrating a Low-Cost Electronic Nose and Machine Learning Modelling to Assess Coffee Aroma Profile and Intensity
Source: Sensors (Basel). 2021 Mar 12;21(6):2016. doi: 10.3390/s21062016 (PMC7998415; doi:10.3390/s21062016)
Supplement: Supplementary file 1 [file sensors-21-02016-s001.pdf]

Table S1. Identified compounds from the gas chromatography mass-spectroscopy analysis showing the mean values of the peak area

Label	RT	Coffee I4	Coffee I5	Coffee I6	Coffee I8	Coffee I9	Coffee I10	Coffee I11	Coffee I12	Coffee I13
**C1**	2.304	1053765^{c} ±291991	1227054^{c} ±120896	2134968^{a} ±137599	1737572^{abc} ±159278	1060691^{c} ±200418	1946113^{ab} ±93434	1627479^{abc} ±236631	1998353^{ab} ±509691	1311814^{bc} ±84050
**C2** ^{NS}	3.763	327810 ±112227	314824 ±44491	437281 ±24534	357825 ±33742	247115 ±95329	353378 ±120312	331812 ±36877	373511 ±98986	216431 ±15910
**C3**	4.157	193841^{bc} ±64614	219241^{bc} ±73080	233863^{a} ±15727	213637^{ab} ±71512	151040^{bc} ±50347	167024^{ab} ±14771	135208^{bc} ±45069	0^{c} ±0	0^{c} ±0
**C4**	4.653	2007006^{c} ±125299	1600609^{d} ±21346	2273003^{c} ±42662	2061787^{c} ±80816	2589969^{b} ±63680	2696908^{b} ±47734	3392361^{a} ±164671	3329210^{a} ±202558	3535933^{a} ±29725
**C5** ^{NS}	5.421	0 ±0	256442 ±85481	256502 ±85501	223321 ±74440	0 ±0	186816 ±62272	323193 ±147590	308720 ±77730	117050 ±4575
**C6**	5.583	120276^{d} ±40092	143614^{bcd} ±50631	162337^{ab} ±10720	144670^{bcd} ±48268	145138^{cd} ±48379	153928^{abc} ±4899	207336^{ab} ±18219	256717^{a} ±58560	165139^{ab} ±8888
**C7** ^{NS}	7.243	1785674 ±595225	1633842 ±30077	1460163 ±11018	1546575 ±37786	1538904 ±30647	1339267 ±16479	1602119 ±44174	1639911 ±50151	1644665 ±22261
**C8** ^{NS}	7.540	218608 ±13837	208291 ±6918	295893 ±1175	255649 ±881	238271 ±10495	338154 ±4798	274664 ±93060	277236 ±35041	300148 ±5095
**C9**	7.626	3414080^{ab} ±379364	4151779^{a} ±123706	3250383^{ab} ±19170	3126573^{ab} ±56987	2637467^{bc} ±28521	3283118^{bcd} ±1101658	1456729^{cde} ±41108	1397122^{e} ±465803	1257113^{de} ±17213
**C10**	8.344	6788562^{a} ±130376	5811678^{bc} ±113125	5981577^{b} ±107428	5740074^{bc} ±115401	6717083^{a} ±124089	5815504^{bc} ±167451	5817353^{bc} ±248483	5466434^{cd} ±261034	5123077^{d} ±98864
**C11**	9.299	0^{b} ±0	0^{b} ±0	0^{b} ±0	0^{b} ±0	0^{b} ±0	130133^{ab} ±43377.67	200858^{ab} ±67425	216329^{a} ±83860.56	139713^{a} ±5511.048
**C12** ^{NS}	9.886	1628381 ±77709	1625811 ±13507	1726694 ±575729	1581779 ±13246	1744358 ±53675	1710289 ±570096	1262429 ±29829	1082072 ±44467	1064173 ±354756
**C13**	9.953	1642057^{ab} ±108712	1601205^{ab} ±41704	1486332^{bc} ±57931	1522647^{bc} ±63778	1516707^{bc} ±21698	1369429^{c} ±39819	1649144^{ab} ±66684	1627378^{ab} ±63504	1712270^{a} ±16342
**C14**	11.043	266466^{a} ±23716	250106^{ab} ±6060	288032^{a} ±11329	269329^{a} ±9663	246102^{b} ±82452	248656^{ab} ±8520	162581^{c} ±54194	0^{c} ±0	0c ±0
**C15**	11.156	608613^{e} ±79344	729479^{d} ±12128	826432^{cd} ±33873	862998^{c} ±16757	1108060^{a} ±24445	986752^{b} ±5260	992259^{b} ±15399	1164075^{a} ±37535	1065226^{ab} ±28400
**C16**	11.249	5855313^{c}	7096012^{a}	6761174^{ab}	6658752^{abc}	6275168^{bc}	6113317^{bc}	3287250^{d}	2906720^{d}	2615782^{d}

		±755535	±61886	±93131	±60520	±218249	±17650	±147179	±36973	±49268
**C17**	11.723	229712^{ef}	242610^{f}	325062^{ef}	317257^{ef}	559170^{cd}	527139^{de}	779232^{bc}	1176831^{a}	844466^{b}
		±76610	±80870	±108363	±5204	±29590	±175713	±12020	±32159	±37002
**C18**	12.046	10478104^{c}	9964917^{c}	12929237^{a}	11960177^{b}	13156807^{a}	13007757^{a}	10262411^{c}	9668104^{c}	9655968^{c}
		±451659	±252543	±178240	±222318	±572930	±219927	±110333	±357730	±17367
**C19**	12.067	1103864^{de}	1044390^{e}	1105476^{de}	1248787^{bc}	1176886^{cd}	1169914^{cd}	1368238^{ab}	1410032^{a}	1367150^{ab}
		±73415	±35653	±10994	±14306	±72959	±13036	±11967	±38821	±27613
**C20**	12.124	1309043^{a}	1254666^{a}	0^{b}	1401484^{a}	0^{b}	0^{b}	0^{b}	0^{b}	0^{b}
		±32036.17	±419422.3	±0	±478359.3	±0	±0	±0	±0	±0
**C21**	12.205	0^{c}	0^{c}	0^{c}	0^{c}	0^{c}	0^{c}	0^{c}	934328^{a}	862015^{b}
		±0	±0	±0	±0	±0	±0	±0	±17226.06	±15494.85
**C22**	12.241	1235885^{bcd}	1199322^{cde}	1352693^{ab}	1344330^{ab}	1380534^{a}	1325751^{abc}	1172956^{de}	1002786^{f}	1101308^{ef}
		±73574	±28636	±24840	±41317	±69217	±14448	±34195	±39424	±13751
**C23**	12.342	492455^{bc}	551638^{c}	666609^{ab}	639165^{ab}	708901^{a}	721076^{a}	568415^{ab}	562243^{bc}	496220^{ab}
		±57187	±183888	±7444	±13599	±25039	±3385	±17041	±9209	±2879
**C24**	12.978	0^{d}	97316^{cd}	0^{d}	0^{d}	154948^{a}	137905^{a}	115030^{ab}	124825^{ab}	109786^{bc}
		±0	±32439	±0	±0	±11942	±3060	±2966	±41677	±4125
**C25**	13.031	168261^{bc}	217799^{ab}	287265^{a}	229958^{ab}	181211^{bc}	214905^{bc}	143650^{c}	146111^{c}	113831^{c}
		±56149	±11106	±3698	±14045	±60421	±74919	±47883	±48704	±38042
**C26**	13.473	0^{b}	416784^{a}	522434^{a}	540592^{a}	712542^{a}	631768^{a}	609928^{a}	652542^{a}	572111^{a}
		±0	±3663	±8281	±6332	±240830	±14545	±203314	±16460	±19816
**C27** ^{NS}	13.556	0	103754	140336	125525	153055	162593	165567	177469	137361
		±0	±34585	±46779	±41842	±4308	±54200	±4119	±59156	±45996
**C28**	13.800	668580^{e}	708023^{de}	860823^{bc}	841317^{bc}	1102637^{a}	948175^{b}	925532^{bc}	850016^{bc}	817329^{cd}
		±74916	±16593	±8276	±6838	±59746	±29835	±19585	±43147	±7418
**C29**	13.888	1003104^{cd}	1098025^{bcd}	1147563^{d}	1392439^{abc}	1310453^{d}	1316760^{cd}	1485687^{abc}	1919063^{ab}	1785775^{a}
		±98905	±24511	±382521	±21768	±436818	±438941	±8317	±43641	±23483
**C30**	13.916	190089^{g}	231318^{fg}	294787^{def}	255760^{efg}	319476^{cde}	357757^{cd}	392785^{bc}	628924^{a}	473549^{b}
		±18433	±17072	±6610	±12071	±17828	±18993	±13225	±76641	±4968
**C31** ^{NS}	14.014	1429050	2419757	3081367	2574165	2437543	3264639	3864495	4519789	3187485
		±584802	±160188	±1027797	±68873	±334795	±163911	±1288809	±795008	±119815
**C32**	14.076	711526^{f}	748129^{ef}	941187^{abc}	891487^{bcd}	987862^{ab}	1057238^{a}	813647^{def}	858396^{cde}	845404^{cde}
		±66405	±67601	±21682	±28184	±37756	±9730	±12197	±58572	±18082
**C33**	14.121	0^{d}	651529^{cd}	0^{d}	583035^{cd}	1247061^{bcd}	1324742^{bcd}	2656167^{abc}	3401537^{ab}	2847172^{a}

		±0	±217940	±0	±232048	±53170	±12422	±885658	±1136186	±84739
**C34**	14.523	0^{c}	134537^{ab}	185623^{a}	168595^{a}	160138^{bc}	174365^{a}	180291^{ab}	141980^{bc}	152018^{a}
		±0	±3057	±7113	±3491	±53379	±14591	±60190	±47327	±3829
**C35**	14.964	0^{b}	108770^{b}	0^{b}	0^{b}	169956^{a}	156408^{a}	134192^{b}	0^{b}	117647^{b}
		±0	±36257	±0	±0	±18442	±3262	±44731	±0	±39216
**C36**	15.160	0^{d}	0^{d}	0^{d}	0^{d}	0^{d}	0^{d}	146981^{c}	214785^{a}	173947^{b}
		±0	±0	±0	±0	±0	±0	±5344	±9926	±4663
**C37**	15.952	0^{c}	0^{c}	867443^{ab}	700442^{c}	807607^{c}	936482^{ab}	981023^{ab}	1141178^{a}	712406^{b}
		±0	±0	±13565	±233481	±269202	±26059	±18901	±211130	±39099
**C38**	15.998	1459498^{d}	1832398^{cd}	2079813^{bc}	1909384^{bcd}	1851550^{cd}	1888357^{cde}	2085829^{bc}	2841068^{a}	2343388^{b}
		±248665	±29718	±39368	±78675	±88584	±160571	±121417	±334340	±52831
**C39**	16.246	0^{c}	153974^{ab}	135732^{b}	0^{c}	161911^{ab}	145174^{b}	0^{c}	134175^{b}	220520^{a}
		±0	±2996	±45265	±0	±1966	±48403	±0	±45169	±9325
**C40**	16.578	0^{b}	0^{b}	0^{b}	0^{b}	0^{b}	0^{b}	133399^{a}	152210^{a}	102739^{b}
		±0	±0	±0	±0	±0	±0	±6791	±50756	±34246
**C41**	16.695	0^{d}	103791^{cd}	163695^{bc}	124257^{cd}	149239^{ab}	211378^{a}	188224^{ab}	230121^{a}	223775^{a}
		±0	±34597	±55224	±41419	±8253	±18481	±4116	±27470	±3598
**C42**	16.746	0^{f}	124204^{e}	157150^{de}	174338^{d}	167661^{de}	186167^{cd}	226569^{bc}	272664^{a}	252841^{ab}
		±0	±5029	±8631	±4775	±17417	±5770	±5980	±37107	±7732
**C43**	17.770	188811^{f}	352948^{ef}	453686^{e}	472696^{e}	758849^{d}	926466^{d}	1895337^{c}	3703017^{a}	3050747^{b}
		±34532	±13245	±9556	±58444	±12275	±41109	±88263	±166000	±81227
**C44**	18.159	196534^{g}	284241^{f}	433956^{e}	447959^{e}	675638^{d}	742902^{d}	836121^{c}	1235111^{a}	1049600^{b}
		±29190	±5727	±18578	±16052	±5652	±33698	±17505	±53823	±16645
**C45**	18.381	126538^{c}	209608^{c}	186368^{c}	207068^{c}	170703^{c}	171157^{c}	211068b^{c}	422079^{a}	321126^{ab}
		±42276	±69887	±6027	±69285	±57444	±57062	±4198	±11735	±7327

$^{a-g}$ Different letters depict the significant differences ($p < 0.05$) between samples (columns) based on the ANOVA and Least Significant Difference *post hoc* test. ^{NS} Non-significant differences.

Table S2. Factor loadings of the main two components of the principal component analysis. Abbreviations of the compounds are found in Table 3; PC1 and PC2: Principal component one and two.

**Compound**	**PC1**	**PC2**
C1	0.07	-0.17
C2	-0.04	-0.10
**C3**	***-0.18***	-0.09
C4	0.18	-0.01
C5	0.09	-0.06
C6	0.17	<-0.01
**C7**	<-0.01	**0.30**
C8	0.10	-0.18
**C9**	***-0.18***	-0.05
C10	-0.14	<0.01
C11	0.18	0.01
C12	-0.18	-0.14
**C13**	0.07	**0.27**
**C14**	***-0.18***	-0.11
C15	0.17	-0.12
C16	-0.18	-0.11
**C17**	**0.20**	0.01
C18	-0.09	-0.27
C19	0.18	0.04
C20	-0.14	0.15
C21	0.17	0.11
C22	-0.15	-0.19
**C23**	-0.04	***-0.30***
C24	0.12	-0.08
C25	-0.13	-0.18
C26	0.13	-0.22
C27	0.14	-0.21
C28	0.06	-0.24
C29	0.19	0.02
C30	0.20	-0.01
C31	0.18	-0.09
**C32**	0.03	***-0.29***
C33	0.19	0.04
C34	0.08	-0.24
C35	0.03	-0.11
C36	0.19	0.11
C37	0.14	-0.19
C38	0.18	-0.02
C39	0.07	-0.08
C40	0.18	0.10
C41	0.17	-0.15
C42	0.18	-0.10
**C43**	**0.20**	0.06
**C44**	**0.20**	-0.03
C45	0.18	0.06

Data in straight bold depict the main compounds that represent the principal components on the positive side of the axis, while data in bold and italics are the compounds that represent the principal components on the negative side of the axis.
